# Neuronal correlates of decisions to speak and act: Spontaneous emergence and dynamic topographies in a computational model of frontal and temporal areas

**DOI:** 10.1016/j.bandl.2013.02.001

**Published:** 2013-10

**Authors:** Max Garagnani, Friedemann Pulvermüller

**Affiliations:** a Medical Research Council, Cognition and Brain Sciences Unit, 15 Chaucer Rd., Cambridge CB2 7EF, United Kingdom; b Brain Language Laboratory, Department of Philosophy and Humanities, Freie University of Berlin, Habelschwerdter Allee 45, 14195 Berlin, Germany

**Keywords:** Voluntary action, Functional specialisation, Free will, Language, Speech, Prefrontal cortex, Neural network, Hebbian learning, Connectivity, Readiness potential

## Abstract

The neural mechanisms underlying the spontaneous, stimulus-independent emergence of intentions and decisions to act are poorly understood. Using a neurobiologically realistic model of frontal and temporal areas of the brain, we simulated the learning of perception–action circuits for speech and hand-related actions and subsequently observed their spontaneous behaviour. Noise-driven accumulation of reverberant activity in these circuits leads to their spontaneous ignition and partial-to-full activation, which we interpret, respectively, as model correlates of action *intention emergence* and action *decision-and-execution*. Importantly, activity emerged first in higher-association prefrontal and temporal cortices, subsequently spreading to secondary and finally primary sensorimotor model-areas, hence reproducing the dynamics of cortical correlates of voluntary action revealed by readiness-potential and verb-generation experiments. This model for the first time explains the cortical origins and topography of endogenous action decisions, and the natural emergence of functional specialisation in the cortex, as mechanistic consequences of neurobiological principles, anatomical structure and sensorimotor experience.

## Highlights

► We simulate emergence of decisions to speak and act in a model of the human brain. ► Action intentions spontaneously emerge due to the reverberation of neuronal noise. ► Spontaneous ignition preferentially occurs in higher-association, multimodal areas. ► Connectivity and learning explain cortical dynamics underlying action decisions. ► Connectivity and learning explain natural emergence of cortical specialisation.

## 1 Introduction

Voluntary action and the ability it entails to decide whether, how, and when to act is a most essential feature of humans. In contrast to responses triggered by an external stimulus, the antecedents of voluntary action lie entirely in internal cognitive processes whose neurobiological underpinnings are poorly understood. Regardless of whether the laws that govern what can be considered free decisions – e.g., deciding whether and when to lift a finger, whether to utter a word and which – are causally determined in a strict sense or rather probabilistic in nature, it is important to delineate the cortical *mechanisms* underlying such self-initiated decisions, asking *how* in the brain the “birth” of a thought carrying an intention to speak or act (“action thought” or “action intention” for short) might occur. No explanation currently exists for such spontaneous thoughts emergence. Ideally, such an explanation should be provided in terms of a mechanistic model based *exclusively* on neurobiological principles, and should elucidate why the brain correlates of action intentions tend to arise first within a specific set of cortical areas and then subsequently spread to others, as experimentally observed (see below). We provide one such explanatory account here. The present model also offers the first neurobiologically based account of the emergence of *functional specialisation* in the human brain, illustrating the precise neural mechanisms by means of which specific cortical areas may spontaneously “take on” specific cognitive processes, such as planning and decision making.

**Fig. 1:**
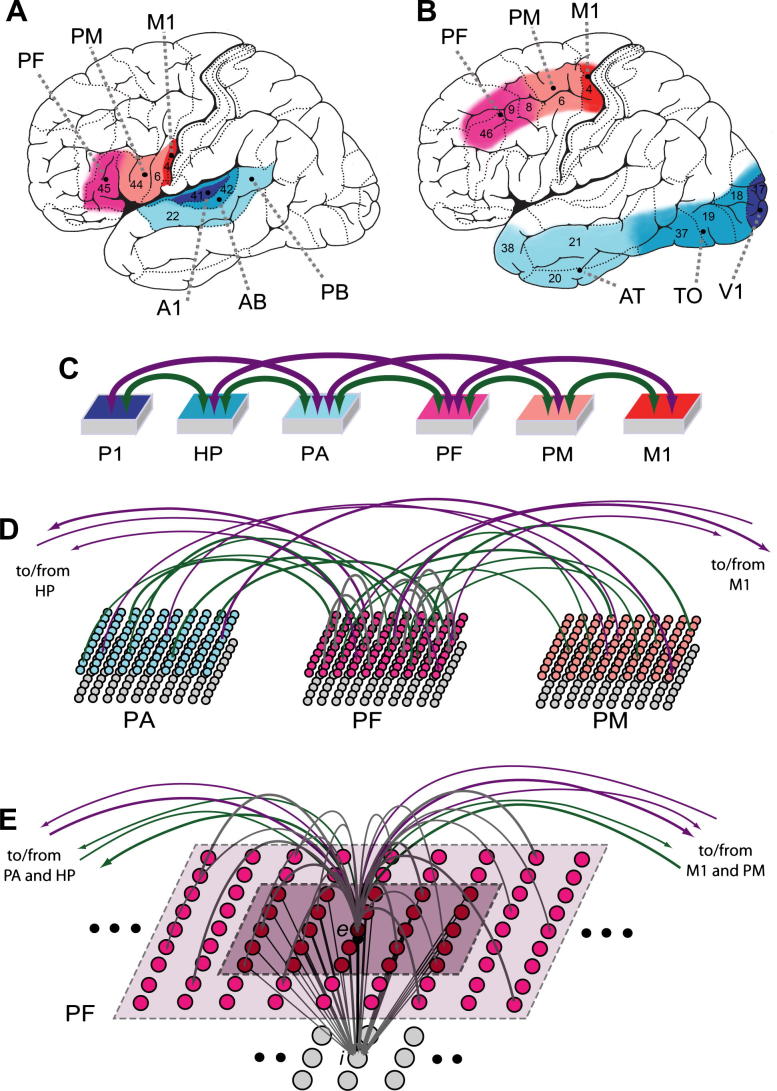
Brain areas, model architecture, and connectivity. **(A)-(B)** Sets of cortical areas modelled. Areas relevant for learning the associations between (A) articulatory movements and resultant sounds – that is, primary auditory cortex, labelled A1 (Brodmann Area 41), auditory belt, labelled AB (BA 42) and parabelt, PB (BA 22), primary motor, or M1 (ventral part of BA 4), premotor, or PM (ventral BA 6 and BA 44) and prefrontal, or PF (BA 45) cortex – and (B) visual stimuli and hand motor actions: primary visual, or V1 (BA 17), temporo-occipital, or TO (including ventral parts of the occipital lobe, BA 18/19, and posterior parts of the middle and inferior temporal gyri, BA 37) and anterior-temporal, or AT (including the temporal pole, BA 38, and middle parts of the inferior and middle temporal gyri, BA 20/21) areas, and, primary motor, M1 (dorsolateral part of BA 4), adjacent premotor, PM (part of BA 6) and prefrontal, PF (parts of BA 8/9/46) cortices, with M1, PM and PF limited to the most lateral parts of the superior, and dorsal parts of the middle, frontal gyri. **(C)** Architecture of the model used for simulating learning of sensorimotor associations and their spontaneous activation. Model areas correspond to cortical areas (as indicated by colour code): primary motor (M1), premotor (PM), prefrontal (PF), and primary perceptual (P1), higher perceptual (HP) and perceptual association (PA) areas. All between-areas connections realised are based on known neuroanatomical links. **(D)** Structure of, and connectivity between, 3 areas of the model. Each area consists of two layers of 25 × 25 excitatory (upper) and inhibitory (lower) graded-response leaky integrator cells exhibiting neuronal fatigue. Between-area connections (green and purple) are sparse, random and topographic (not illustrated). **(E)** Illustration of connectivity of a single excitatory cell (labelled “*e*”). Within-area excitatory links (in grey) to and from *e* are limited to a local (19 × 19) neighbourhood (light-coloured area). Mutual inhibition between *e* and its neighbours is implemented by underlying cell “*i*”, which receives input from a 5 × 5 neighbourhood (dark-coloured area) and projects back to *e*, inhibiting it (*e*’s neighbours are similarly inhibited by *e*). Each pair (*e,i*) of cells represents clusters of pyramidal cells and interneurons within the same cortical column of approximately 0.25 mm^2^ size (containing ∼25,000 neurons (Braitenberg & Schüz, 1998)).

Several studies have addressed aspects of the problem of spontaneous emergence of action thoughts. When subjects choose freely the time point of a pre-defined action (typically a button press), a so-called “readiness potential” (RP) or “*Bereitschaftspotential”* emerges (Kornhuber and Deecke, 1965, Shibasaki and Hallett, 2006), whose cortical generators can be localised. More precisely, converging evidence from single-unit studies in the monkey (Romo & Schultz, 1987), neurometabolic imaging studies in man (Wiese et al., 2004) and epicortical recordings in patients (Ikeda & Shibasaki, 2003) enable the identification of plausible RP cortical sources in terms of a cascade of activations, starting in anterior prefrontal areas at about 1–1.5 s before movement onset and proceeding rostro-caudally, as follows: (1) initial (bilateral) activity in prefrontal cortex, putatively underlying the emergence of an intention to act, followed by (2) sources in supplementary motor and dorsal premotor areas, leading to (3) lateralised activation in dorsolateral (hand-specific) primary motor cortex (Haggard, 2008). Similar results have been found by studies investigating the origins of the RP preceding speech (Deecke et al., 1986, McArdle et al., 2009), with cortical generators localised more ventrally than those identified for distant limb movements, i.e., in inferior prefrontal, adjacent premotor, and articulatory (mouth-specific) primary motor areas. Together, these results suggest the existence of (at least) two parallel systems for the planning and execution of voluntary movement (see red-shaded areas in Fig. 1, panels A-B): (i) one for speech, located in the prefrontal (PF), premotor (PM) and primary motor (M1) cortices of the inferior frontal gyrus Fig. 1A; and (ii) one for hand and finger movements, involving the dorsolateral parts of the middle and ventrolateral parts of the superior frontal gyri Fig. 1B.

**Fig. 2:**
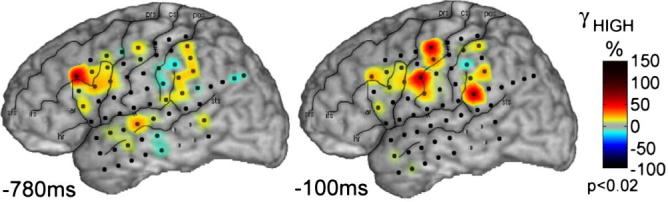
Example of cortical activation during a verb generation task. Topographies of high-gamma (70–160 Hz) analytical amplitude of the electro-corticogram of an epileptic patient showing sequential activation in prefrontal (left) and motor (right) areas shortly before production (at 780 and 100 ms pre-response) of an appropriate verb in response to auditory presentation of a noun (*adapted from* (Edwards et al., 2010), their Fig. 3).

In the language domain, an approach frequently adopted to investigate the origins of spontaneous, internally driven activation of thoughts involves the use of “underdetermined” tasks, requiring participants to choose and verbalize one amongst a set of possible candidate concepts previously primed within the semantic system. Similarly to the studies on speech-elicited RP, these tasks (e.g., verb generation, sentence completion, word generation) have been found to reliably activate the left inferior prefrontal cortex (Broca’s area), but, in addition, posterior–superior temporal cortex (Wernicke’s area) (Raichle et al., 1994, Thompson-Schill et al., 1997, Wise et al., 1991). Fig. 2 (adapted from (Edwards et al., 2010)) depicts an example of cortical activation occurring shortly before speech production in a verb generation task.

Readiness-potential and verb-generation tasks fail to capture all the characteristics of entirely “free” decisions, focussing in one case on the time point of the action decision and in the other on the selection decision among stimulus-related alternatives. However, they provide converging evidence on the brain basis of the aspects of decision processes they monitor: inferior prefrontal and posterior–superior temporal areas are involved first when speech-action decisions are taken, while dorsolateral prefrontal cortex is the earliest site of hand-related action thought emergence. Nevertheless, neither verb generation nor RP studies have so far provided a mechanistic explanation of *how*, at the level of cortical circuits, an intention to act might actually emerge, or addressed the question of *why* such spontaneous processes originate within the prefrontal and “higher” association areas identified (and not, e.g., in primary motor areas, which control the respective movements).

To address these issues, we simulated learning and spontaneous emergence of action thoughts in a neural-network model that closely replicates neurophysiology, structure and connectivity of relevant primary sensorimotor, secondary, and higher association areas in the frontal and temporal cortex (see Fig. 1, panels A–C). By “action thought” here we mean the activation (“ignition”) of a circuit of strongly and reciprocally connected cells binding together perception and action patterns, referred to as *cell assembly* (CA) (Hebb, 1949). Our previous studies have demonstrated that CAs can emerge in multi-area networks by means of purely Hebbian learning mechanisms and, once developed, behave as non-linear functional units with two stable states (“on” and “off”), which become fully active (“on”) whenever a critical amount of activation – conveyed by external or internal input – is present in their circuits (Garagnani, Wennekers, & Pulvermüller, 2008). Given that CAs are perception–action circuits associating sensory and motor neural patterns that are re-activated when the CA ignites, here we take full CA activation to be the model-correlate of the *conscious perception of an action thought*.

To simulate non-stimulus driven, endogenous perception–action CA circuit ignition in the relevant sensorimotor systems we took a network mimicking structure and connectivity of these systems (see Fig. 1C–E) and in which such circuits had previously formed as a result of Hebbian learning and sensory-motor stimulation (Garagnani et al., 2008), and recorded its spontaneous activity in absence of any input, while activity was driven purely by noise (simulating spontaneous neuronal firing). All structural and functional properties of the model reproduce connectivity features and cellular-level phenomena that have been observed pervasively in the cortex, including local (lateral) inhibition, patchy, sparse and topographical connectivity (Braitenberg and Schüz, 1998, Kaas, 1997), activation spreading and summation, noise, and long-term synaptic plasticity (Malenka & Bear, 2004). Solely on the basis of these neurobiological principles and anatomical constraints the model provides a mechanistic explanation of why intentions to act preferentially originate from a specific set of brain areas.

A number of computational models of decision making and action selection in the human brain has been proposed in the past (Bogacz and Gurney, 2007, Botvinick and Plaut, 2004, Frank and Claus, 2006, Pfeiffer et al., 2010, Simen et al., 2006, Usher and McClelland, 2001). None of these, however, has focussed on the spontaneous emergence of stimulus-independent, voluntary action, or on explaining the characteristic topography of the cortical sources believed to underlie this process. Most relevant here is the ground-breaking work by Deco and collaborators (Deco and Rolls, 2005, Deco and Rolls, 2006, Deco et al., 2009, Deco et al., 2007, Rolls et al., 2010a, Rolls et al., 2010b), who investigated attractor networks of interacting integrate-and-fire neurons exhibiting the ability to spontaneously “jump” out of a resting state into one of two high firing-rate stable states as a result of background noise and sensory “bias”. This work speaks directly to cognitive-psychological proposals about decision generation based on probabilistic biased-competition models (Duncan, 1980) of decision making. The neural-networks described therein, however, do not attempt to replicate in details the connectivity features of the modelled primary sensory and motor, and higher, cortical areas (e.g., the sparse and patchy connections typical of the cortex). Indeed, while this pioneering work well illustrates the principles and stochastic mechanisms that may underlie decision-making in the human brain, it cannot (and, we believe, was not meant to) explain the cortical topography revealed by the neuroimaging and neurophysiological experiments on volitional action initiation reviewed earlier, object of the present study.

## 2 Materials and methods

In this work, we take the basic building blocks of internal cognitive representations to consist of widely distributed networks of strongly connected neurons, referred to as *cell assemblies* (CAs) (Braitenberg, 1978, Hebb, 1949, Palm, 1982). CAs constitute “memory circuits” that bind together frequently co-occurring perception and action activation patterns (Fuster, 2006) and which gradually emerge in the cortex as a result of experience, by means of Hebbian associative learning mechanisms. More precisely, repeated co-activation of specific sets of neurons in primary sensory and motor cortices leads to the strengthening of the synaptic links between them, and to the formation of perception–action circuits distributed over sensory, motor and mediating “higher” areas (Fuster, 2001, Fuster, 2003). Note that, in the present approach, the intrinsic connectivity between relevant sensory, motor, and related higher cortical areas (discussed below) is *key* to the formation (and hence distribution) of CAs.

### 2.1 Structure and function of the relevant brain areas

In view of the experimental studies reviewed in the Introduction, we focus on modelling CA emergence and spontaneous CA activation dynamics in two specific sensorimotor brain systems, namely:

(A) the left perisylvian areas in the superior temporal and inferior frontal gyri (Fig. 1A), involved in (and repeatedly co-activated during) early stages of language acquisition and spoken language processing (Fadiga et al., 2002, Fry, 1966, Pulvermüller, 1992, Pulvermüller, 1999, Zatorre et al., 1996), and
(B) the left ventral visual “what”-system and the motor system (Fig. 1B) relevant for processing, respectively, visual object identity (Ungerleider and Haxby, 1994, Ungerleider and Mishkin, 1982) and manual actions (Deiber et al., 1991, Dum and Strick, 2002, Dum and Strick, 2005, Lu et al., 1994).

Documented anatomical connections existing between the areas of left perisylvian system (A) include: (i) reciprocal links between cortically adjacent areas in both the superior temporal (BA 41, 42, 22)(Kaas and Hackett, 2000, Pandya, 1995, Rauschecker and Tian, 2000) and inferior-frontal (ventral BA 4, 6/44, 45/46) gyri (Pandya and Yeterian, 1985, Young et al., 1995, Young et al., 1994) (see also (Pulvermüller, 1992)), (ii) long-distance cortico-cortical connections bridging inferior prefrontal (BA 45) and posterior–superior temporal (BA 22) areas (i.e., PF and PB in Fig. 1A) mainly via the arcuate fascicle and the extreme capsule (Catani et al., 2005, Makris and Pandya, 2009, Makris et al., 1999, Parker et al., 2005, Petrides and Pandya, 2001, Romanski et al., 1999b), and (iii) “jumping” links (i.e., between areas that are not adjacent in the model – see purple arrows in Fig. 1C), consisting of long-distance cortico-cortical connections reciprocally linking auditory parabelt (BA 22) with premotor (BA 6/44) (Glasser and Rilling, 2008, Saur et al., 2008, Saur et al., 2010) and belt (BA 42) with prefrontal (BA 45) (Kaas and Hackett, 2000, Petrides and Pandya, 2009, Romanski et al., 1999a) areas, as well as non-adjacent areas in superior-temporal (BA 41 with BA 22) and inferior-frontal (BA 45 with BA 4) (Pandya and Yeterian, 1985, Young et al., 1994) gyri.^1^

In the frontal lobe (refer to system (B), red-shaded areas in Fig. 1B), the hand/arm motor area M1 (in BA 4), adjacent PM (BA 6) and more rostral PF (BA 8/9/46) areas are reciprocally connected (Arikuni et al., 1988, Dum and Strick, 2002, Dum and Strick, 2005, Lu et al., 1994, Pandya and Yeterian, 1985, Rizzolatti and Luppino, 2001), with cortico-cortical links documented between PF and M1 (Guye et al., 2003, Young et al., 1995). The primary visual, V1 (BA 17), temporo-occipital, TO (occipital lobe, inferior parts of BA 18/19, and posterior parts of the inferior and middle temporal gyri, BA 37) and anterior-temporal, AT (temporal pole, BA 38, and middle parts of the middle and inferior temporal gyri, BA 20/21) areas of the same system (B) (Fig. 1B, blue-shaded areas) are reciprocally connected (Distler et al., 1993, Nakamura et al., 1993). Direct (jumping) links from V1 to anterior temporal (AT) regions via the inferior longitudinal fascicle have also been documented (Catani et al., 2003, Wakana et al., 2004). Neuroanatomical (Ungerleider et al., 1989, Webster et al., 1994), inactivation (Bauer and Fuster, 1976, Chafee and Goldman-Rakic, 2000, Fuster et al., 1985) and lesion (Eacott and Gaffan, 1992, Parker and Gaffan, 1998a, Parker and Gaffan, 1998b) studies in the monkey indicate the presence of direct connections (via the uncinate fascicle) also between anterior-temporal and prefrontal cortices (AT and PF in Fig. 1B). Evidence suggests the presence of direct links (via the external capsule) also between temporo-occipital (TO) and prefrontal areas (PF) (Makris and Pandya, 2009, Pandya and Barnes, 1987, Seltzer and Pandya, 1989) and between anterior/middle temporal (AT) and premotor (PM) areas (Rizzolatti & Luppino, 2001).

In the perisylvian system (A), correlated auditory-articulatory activity in inferior motor cortex (articulator representation) and consequent auditory-evoked superior temporal area activity leads, in presence of Hebbian learning mechanisms, to linkage of word-articulation neural patterns with their corresponding acoustic–phonetic patterns (Fadiga et al., 2002, Pulvermüller, 1999, Pulvermüller, 2001, Watkins et al., 2003, Wilson et al., 2004, Zatorre et al., 1996) (see (Pulvermüller & Fadiga, 2010) for a review). Similarly, Hebbian mechanisms acting in system (B) support the development of CAs linking visual stimuli to hand/arm movements, as required, for example, to pair up visual identity of objects with associated reaching and grasping actions (Arbib, 1981, Gallese et al., 1996, Grafton et al., 1997, Grezes and Decety, 2002), or, more generally, to acquire conditional visuomotor associations arbitrarily mapping visual features of objects to specific actions (Eacott and Gaffan, 1992, Muhammad et al., 2006, Toni et al., 2001, White and Wise, 1999).

### 2.2 Structure and function of the model

Because systems (A) and (B) exhibit analogous structure and high-level connectivity (see above and Fig. 1A and B), the same network architecture can be used to model both. This is possible because the local connection structures implemented in the model reflect connectivity features that are shared by sensory and motor systems of the mammalian brain (Braitenberg and Schüz, 1998, Douglas and Martin, 2004); moreover, all functional mechanisms realised at cellular-level (e.g., neuronal adaptation, noise, synaptic plasticity – see Eqs. (1), (2), (3), (4.1), (4.2), (5) below) reflect neurobiological phenomena that are found pervasively in the cortex (Kandel et al., 2000, Rauschecker, 1999). In view of this, in Fig. 1C we adopt a generic labelling of the model areas, as indicated by the colour code used there.

Each model area consists of two layers of 625 excitatory and 625 inhibitory cells (see Fig. 1D–E)^2^. Each excitatory cell represents a cluster of cortical neurons (pyramidal cells), and the underlying inhibitory cell models the cluster of inhibitory interneurons situated within the same cortical column (Eggert and van Hemmen, 2000, Wilson and Cowan, 1972). The state of each cell *x* is uniquely defined by its membrane potential *V*(*x,t*), representing the average of the sum of all (excitatory and inhibitory) postsynaptic potentials acting upon neural pool (cluster) *x* at time *t*, and governed by the following equation:

(1) $$\tau \cdot \frac{\mathrm{dV}(x\text{,}t)}{\mathrm{dt}}=-V(x\text{,}t)+k_{1}(V_{\mathrm{In}}(x\text{,}t)+k_{2}\eta (x\text{,}t))$$

where *V_In_*(*x,t*) is the net input to cell *x* at time *t* (sum of all inhibitory and excitatory postsynaptic potentials – I/EPSPs; inhibitory synapses are given a negative sign – plus a constant “baseline” term *V_b_*), *τ* is the membrane’s time constant, *k*_1_, *k*_2_ are scaling constants and *η*(*x*,*t*) is a white noise process with uniform distribution over [−0.5,0.5].^3^ Time is in arbitrary units. Cells produce a graded response that represents the average firing rate of the neuronal cluster they model; in particular the output (transformation function) of an excitatory cell *x* at time *t* is:

(2) $$O(x\text{,}t)=\left\{\begin{array}{cc} 0 & \text{if}\;V(x\text{,}t)\leq \varphi \\ \left(V(x\text{,}t)-\varphi \right) & \text{if}\;0<(V(x\text{,}t)-\varphi )\leq 1\\ 1 & \text{otherwise} \end{array}\right)$$

*O*(*x,t*) represents the average firing rate (number of action potentials per time unit) of cluster *x* at time *t*; it is a piecewise-linear sigmoid function of the cell’s membrane potential *V*(*x,t*), clipped into the range [0, 1] and with slope 1 between the lower and upper thresholds *φ* and *φ + *1. The output *O*(*x,t*) of an inhibitory cell is 0 if *V*(*x,t*) < 0, and *V*(*x,t*) otherwise. In excitatory cells, the value of the threshold *φ* in Eq. (2) varies in time, tracking the recent mean activity of the cell so as to implement a simple version of neuronal adaptation (Kandel et al., 2000) (higher activity leads to a higher threshold). More precisely:

(3) φ(x,t)=α·ω(x,t)

where *ω*(*x*,*t*) is the time-average of the cell’s recent output and *α* is the “adaptation strength” (see Appendix A for the exact parameter values used in the simulations).

For an excitatory cell *x*, the approximate time-average *ω*(*x*,*t*) of its output *O*(*x,t*) is estimated by integrating the linear differential equation Eq. (4.1) below with time constant *τ_A_*, assuming initial average *ω*(*x*,0) = 0:

(4.1) $$\tau_{A}\cdot \frac{d\omega (x\text{,}t)}{\mathrm{dt}}=-\omega (x\text{,}t)+O(x\text{,}t)$$

Local (lateral) inhibitory connections (see Fig. 1E) and area-specific inhibition are also implemented, realising, respectively, local and global competition mechanisms (Duncan, 2006) and preventing activation from falling into non-physiological states (Braitenberg & Schüz, 1998). More formally, in Eq. (1) the input *V_In_*(*x,t*) to each excitatory cell of the same area includes an area-specific (“global”) inhibition term *k*_S_·*ω_S_*(*x*,*t*), which is subtracted from the total sum of the I/EPSPs postsynaptic potentials *V_In_* in input to the cell, with *ω_S_*(*x*,*t*) defined by:

(4.2) $$\tau_{S}\cdot \frac{d\omega_{s}(x\text{,}t)}{\mathrm{dt}}=-\omega_{s}(x\text{,}t)+\underset{x\in \mathrm{area}}{\sum }O(x\text{,}t)$$

The low-pass dynamics of the cells (Eqs. (1), (2), (4.1), (4.2)) are integrated using the Euler scheme with step size Δ*t*, where Δ*t* = 0.5 (in arbitrary time units).

Excitatory links within and between (possibly non-adjacent) model areas are random and limited to a local (topographic) neighbourhood; weights are initialised at random, in the range ]0, 0.1]. The probability of a synapse to be created between any two cells falls off with their distance (Braitenberg & Schüz, 1998) according to a Gaussian function clipped to 0 outside the chosen neighbourhood (a square of size *n = *19 for excitatory and *n *= 5 for inhibitory cell projections). This produces a sparse, patchy and topographic connectivity, as typically found in the mammalian cortex (Amir et al., 1993, Braitenberg and Schüz, 1998, Douglas and Martin, 2004, Kaas, 1997).

The Hebbian learning mechanism implemented simulates well-documented synaptic plasticity phenomena of long-term potentiation (LTP) and depression (LTD), believed to play a key role in experience-dependent plasticity, memory and learning (Malenka and Bear, 2004, Rioult-Pedotti et al., 2000). In particular, the learning rule is an implementation of the Artola–Bröcher–Singer model of LTP/LTD (Artola et al., 1990, Artola and Singer, 1993). In the model, we discretized the continuous range of possible synaptic efficacy changes into two possible levels, +Δ*w* and −Δ*w* (with Δ*w* << 1 and fixed). We defined as “active” any link from an excitatory cell *x* such that the output *O*(*x,t*) of cell *x* at time *t* is larger than *θ_pre_*, where *θ_pre_* ∈ ]0,1] is an arbitrary threshold representing the minimum level of presynaptic activity required for LTP (or LTD) to occur. Thus, given any two cells *x* and *y* connected by a synaptic link with weight *w_t_*(*x,y*), the new weight *w_t_*_+1_(*x,y*) is calculated as follows:

(5) $$w_{t+1}(x\text{,}y)=\left\{\begin{array}{ccc} w_{t}(x\text{,}y)+\Delta w & \;(\mathrm{LTP}) & \text{if}\;O(x\text{,}t)\geq \theta_{\mathrm{pre}}\;\text{and}\;V(y\text{,}t)\geq \theta_{+}\\ w_{t}(x\text{,}y)-\Delta w & \;(\mathrm{LTD}) & \text{if}\;O(x\text{,}t)\geq \theta_{\mathrm{pre}}\;\text{and}\;\theta_{-}\leq V(y\text{,}t)<\theta_{+}\\ w_{t}(x\text{,}y)-\Delta w & \;(\mathrm{LTD}) & \text{if}\;O(x\text{,}t)<\theta_{\mathrm{pre}}\;\text{and}\;V(y\text{,}t)\geq \theta_{+}\\ w_{t}(x\text{,}y) & \left(\mathrm{no}\;\mathrm{change}\right) & \mathrm{otherwise} \end{array}\right)$$

Specific parameter values used in the simulations are provided in Appendix A.

### 2.3 Materials

We built a set of 12 to-be-memorised sensorimotor pattern pairs; each pattern pair consists of two (one sensory and one motor) neural configurations, identifying 20 cells in area P1 (representing a perceptual – “auditory” or “visual” – stimulus) and 20 cells in M1 (representing an associated motor – “articulatory” or “manual” – pattern). Note that 20 cells constitute only 3.2% of the total number of cells of one area. The patterns overlap to a degree, to reproduce the corresponding overlap existing between motor and perceptual features of real action schemata. We took a “tabula rasa” network (i.e., one in which all links were initialised at random) having the architecture shown in Fig. 1C, and “taught” it the predefined set of sensory-motor pair associates. More precisely, the training consisted of presenting each of the 12 pattern pairs to the network 15,000 times (in random order). Each presentation involved activating^4^, for 16 simulation time-steps, the cells in P1 and M1 specified by one pair, and was followed by an inter-stimulus interval (ISI) of variable length during which spreading of activation and resultant changes in the synaptic weights (i.e., learning) took place. As shown in our previous work (Garagnani et al., 2007, Garagnani et al., 2009, Garagnani et al., 2008), this training process leads to the spontaneous emergence of pair-specific distributed sensorimotor cell assembly (CA) circuits in the network, each CA linking one (and only one) of the twelve P1 patterns to its corresponding pair associate in M1, in such a way that presentation of just the sensory (or just the motor) component will cause the entire CA to “light up”, leading to the re-activation of the associated pattern in M1 (or P1).

### 2.4 Operative definition of CA ignition

To investigate the emergence of spontaneous CA activation in the network, the model correlate of self-initiated action intentions, we continuously recorded the activity of a trained network for 20,000 simulation steps (roughly equivalent to 400 s. of real time)^5^ while its activity was driven solely by uniform noise (i.e., in absence of any input stimulus). As indicated by Eq. (1), noise is an inherent property of each model cell, intended to mimic the spontaneous activity (baseline firing) of real neurons. This is simulated by a white noise process *η* (identical for all cells) which, at each time step, generated – for each different cell – a random number in the interval [0.5,0.5], with uniform distribution. This value (adequately scaled) is added to the input *V_In_* (see Eq. (1)) that the cell is currently receiving from other cells as well as from external stimuli.^6^ Therefore, noise was present in all areas and in equal amounts (on average).

Network dynamics were documented by recording (in time and for each cortical area) the total number of (excitatory) CA cells^7^ that exhibited output value above a specified threshold *θ* ∈ [0,1]. The threshold *θ* was input- and area-specific, and defined as follows: (i) during training, for each of the 12 input patterns the time-average of the network response to a pattern was computed by averaging all the (excitatory) cells’ output over the 15 time-steps that followed each pattern presentation; (ii) for each pattern *w* and area A, the area-specific threshold *θ* was defined as a given fraction of the maximal single-cell’s average response to input *w*; more formally, $\theta_{w}(A)=\gamma \cdot \max_{x\in A}\overset{¯}{O{\left(x\text{,}t\right)}_{w}}$, where ${\overset{¯}{O(x\text{,}t)}}_{w}$ is the average input-specific response of cell *x* to pattern *w*, and *γ* ∈ [0,1] is a constant; (iii) a cell *x* in area *A* was considered part of the CA for *w* if and only if it exhibited above-threshold average response to *w*, i.e., if ${\overset{¯}{O(x\text{,}t)}}_{w}\geq \theta_{w}(A)$. As different values of *γ* lead to different CA sizes, for the statistical analysis of the CA dynamics (see below) we used *γ* = 0.50; this value was chosen on the basis of simulations with networks having a similar architecture to the present one (Garagnani et al., 2008), showing a relatively invariant CA size for values of *γ* between .1 and .7.

To study the dynamics of spontaneous CA ignition we identified all episodes of CA activation that occurred during the period of spontaneous network activity recorded. A CA was considered active if and only if at least 50% of its cells were firing above threshold *θ* (where *θ* was area- and pattern-specific, as just described).

### 2.5 Analysis of ignition dynamics

Using the definition (iii) given in the previous section we measured average CA size (number of CA cells) per cortical area across different values of *γ* (*γ* = .01, .02, .04, .05, .1, .2, .4, .5). This was done in order to control for any scaling effects (introduced by the presence of different numbers of CA cells in different areas) which may confound the results, and to be able to reveal true effects of area on the spontaneous CA activation dynamics.

The average time-course of spontaneous ignition of each CA (hereafter called “average spontaneous ignition”, ASI) was obtained by averaging together all events (“trials”) of spontaneous activation of the same CA. A trial was 40 time-steps long, starting 10 steps before spontaneous CA activation time (the earliest time-step of the ignition episode at which the CA was active, as defined earlier). For each CA, we computed six different area-specific ASIs. Only and all those CAs which spontaneously ignited at least once during the recorded time (11 out of the 12 learnt) were used in the analysis. For each area, we computed and plotted the average number of CA cells active above threshold by averaging over the 11 different ASIs. We also computed the normalised ASIs – obtained by dividing each (area-specific) ASI by the respective CA size in that area – and their average, thus obtaining the average fraction (%) of active CA cells per cortical area. To statistically test for the presence of different spontaneous ignition time-courses in different cortical areas, we investigated more closely the CA dynamics during a critical 6-step-long time window with origin (time 0) set to the last step at which the average number of active CA cells was still zero (baseline) in all areas. For this interval, we extracted the area-specific activation data from the 11 (normalised) ASIs and subjected these values to repeated-measure analyses of variance (ANOVAs). More precisely, for each of the six time-steps of interest we ran a separate 2-way ANOVA with factors “centrality” (3 levels; *primary* = {P1, M1}, *secondary* = {HP, PM}, and *central* = {PA, PF}) and “frontality” (2 levels; *posterior* = {P1, HP, PA}, *anterior:* = {M1, PM, PF}). Note that this nomenclature, used in the remainder of the paper, takes a model-centred (as opposed to brain-centred) view, in that areas that appear in the periphery of the network (i.e., primary sensory, P1, and motor, M1 – see Fig. 1C) are defined here as “peripheral”, and areas that occupy the centre of the architecture (i.e., PA, PF) are referred to as “central” (in spite of the corresponding cortices being localised most laterally and anteriorly in neuroanatomical terms).

## 3 Results

**Fig. 3:**
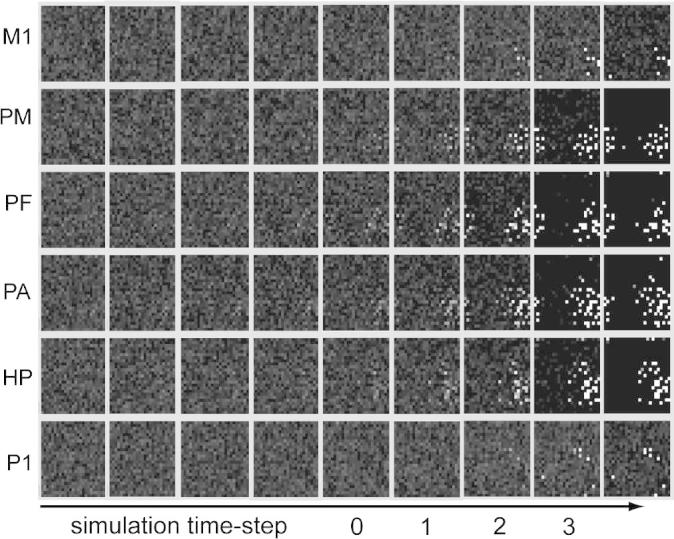
Spontaneous CA ignition. Consecutive snapshots of network activity (from left to right) taken during a typical episode of spontaneous CA activation (i.e., in absence of any input stimulus). Each column depicts activity within the network at a specific time point. Within a column, each square illustrates activity within a specific area; within each area, one “dot”, or pixel, corresponds to an excitatory cell. Brighter pixels indicate cells exhibiting higher firing rates. Preliminary traces of reverberant activity within CA circuits in areas PF and PA can be visually identified already at time −4; from there, CA ignition appears to gradually spread to secondary, and then primary, areas.

During the recorded period of activity (20,000 simulation steps) we observed 197 episodes of spontaneous action-thought circuit ignition, involving 11 different CAs, which activated in random order (the remaining one CA was not observed to ignite spontaneously). Each CA activation was followed by a (spontaneous) de-activation, an effect brought about by the network self-regulation mechanisms (global and local inhibition). Visual inspection of these episodes (e.g., see Fig. 3) suggested that the very first traces of spontaneous reverberant activity within CA cells (CA ignition) emerged preferentially in the “central” areas of the network (PF, PA), corresponding to prefrontal and higher-association cortices in temporal areas, subsequently spreading “outwards” as a cascade of activations proceeding from “central” to secondary (or premotor) and from secondary to primary (motor and sensory) areas.

**Fig. 4:**
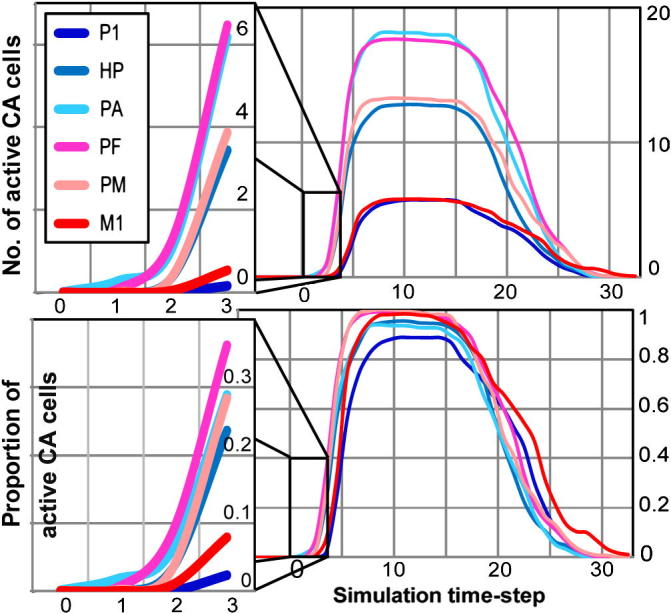
Dynamics of spontaneous CA activation. Right: Time-courses of area-specific average CA activations for the six different cortical areas modelled. Left insets: dynamics of the first few time steps of spontaneous CA ignition. Note the earlier (time-step 1) rise of CA activity in the two central areas (PA, PF: purple and cyan curves), followed by secondary (HP, PM: step 2) and primary (P1, M1: step 3) areas. Top: CA activation quantified as total number of active CA cells per area. Central areas exhibit larger numbers of active CA cells than secondary and primary ones due to CA distribution. Bottom: normalised CA activation data (to remove the confounding effects of CA size, top graphs values are divided by area-specific CA size). While the maximal CA activation levels are now comparable (right), the two central areas still exhibit earlier activation than secondary and primary areas (left).

Average CA size (number of CA cells per area) was 15.4 (P1), 25.1 (HP), 28.4 (PA), 28.5 (PF), 25.8 (PM), 14.9 (M1), indicating a symmetrical distribution of CA-cells around the “centre” of the network, with larger numbers in central than in secondary – and in secondary than in primary – areas. The overall time-course of spontaneous CA activation for the different areas is illustrated in Fig. 4. The graphs plot the area-specific numbers of active CA cells (top: absolute values; bottom: normalised values) against simulation time. All areas exhibit similar profiles, with spontaneous CA activation consisting of three main phases: ignition (time-step −5 to about 5), sustained reverberant activity (from steps 6–7 to ∼15) and deactivation (from ∼15 to ∼30). However, different areas exhibit different numbers of active CA cells during the sustained period, with the two central areas (PA, PF) showing the largest numbers, followed by the two secondary (HP, PM) and two primary ones (P1, M1). This result is a consequence of the cortical distribution of CA cells, described above.

Although the top-left inset of Fig. 4 suggests that CA activity develops first in central areas, this effect could be entirely explained by the larger number of CA cells in these areas. To remove this confound, data were normalised, i.e., divided by area-specific CA size. Even after normalisation (lower plots of Fig. 4), areas PA and PF still appear to exhibit above-baseline CA activity before all others (time-step 1), followed by secondary (step 2) and then by primary (step 3) areas. The distribution of CA neurons over the different areas is therefore insufficient to explain the early emergence of CA activation in the central areas.

**Fig. 5:**
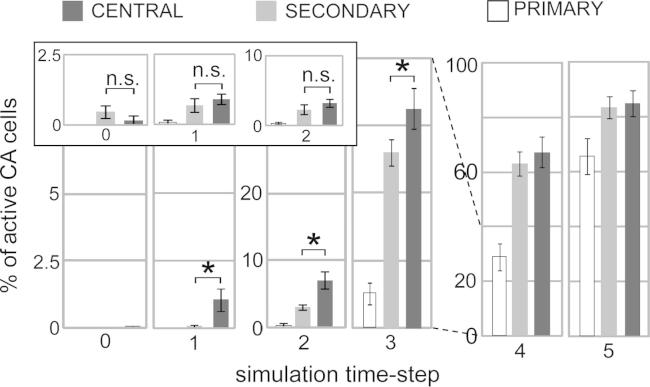
Dynamics of early spontaneous CA ignition. Average portion (%) of CA activation per pairs of cortical areas (Primary = P1, M1; Secondary = PA, PM; Central = HP, PF) during the initial six time-steps. Error bars indicate standard error. Main plots: activity reaches significance first in the central areas (time 1), when secondary areas still exhibit only baseline activity. Secondary areas are first active at time 2, followed by primary areas at time 3. Note the different scales used for the leftmost (times 1–2), middle (3–4) and rightmost (5–6) pairs of plots. Inset: results obtained after removing the “jumping” links (purple arrows in Fig. 1C) from the model. Note that central and secondary areas still become active before primary ones (time-step 1), but, unlike in the “fully connected” model, their activations no longer differ during any of the early CA ignition steps.

The statistical analysis fully confirmed these observations. Each of the five ANOVAs that we ran on the normalised data from time-steps 1 to 5 revealed a main effect of centrality (*F*-values ranged from a minimum of *F*(1,10) = 5.12 at time 1 (*p* < .05) to a maximum of *F*(2,20) = 79.1 (*p* < .00001) at time 4), confirming stronger activity in the central than in the secondary areas already at time 1. Fig. 5 plots the normalised data for the critical time-steps only (time steps 0–5) after grouping them by centrality level (i.e., collapsing data within each pair of *primary*, *secondary*, and *central* areas). Planned comparisons confirmed that CA activation reaches significance first in the central areas (PF, PA) at time-step 1 (*F*(1,10) = 6.37, *p* < .05), when secondary areas are not yet above baseline (*F*(1,10) = 1.2, *p*>.2). These become active later, at time-step 2 (*F*(1,10) = 43.1, *p* < .0001), while activation in primary areas reaches significance last, at time-step 3 (*F*(1,10) = 10.6, *p* < .01). As highlighted in the bar plot, central areas are less active than secondary ones during time-steps 1–3 (*F*-values in the range *F*(1,10) = 5.12 (*p* < .05) to *F*(1,10) = 16.7 (*p* < .005)); similarly, primary are less active than secondary areas between time-steps 2 and 5 (*F*-values from *F*(1,10) = 16.0, *p* < .005, to *F*(1,10) = 226.7, *p* < .000001).

In a further simulation, we repeated the above experiment after removing all the between-area “jumping” links (purple arrows in Fig. 1C) from the model. As before, we observed spontaneous CA ignitions occur in the network (although only 6 of the 12 learnt CAs ignited). Data from this simulation are plotted in Fig. 5, top-left inset. Planned comparisons indicate that, in the altered network, ignition begins both in central and secondary areas: both of these are active at time step 1 (*F*(1,5) = 7.58, *p* < .02 and *F*(1,5) = 22.73, *p* < .0005), when activation in primary areas is still not significant (*F*(1,5) = 1, *p* *>* .1). Primary areas become active only at time step 5 (*F*(1,5) = 3.79, *p* < .05). Activations in secondary and central areas do not differ during time-steps 0, 1 or 2 (*F*(1,5) = 1.57 (*p* *>* .2), *F*(1,5) = 0.49 (*p* *>* .5), and *F*(1,5) = 3.31 (*p* *>* .1), respectively). Moreover, activity in *both* secondary and central areas is stronger than in primary ones already at time-step 2 (*F*(1,5) = 8.68, *p* < .02, and *F*(1,5) = 22.9, *p* < .0005, respectively). Finally, in one last simulation we repeated the same experiment after removing the spontaneous neuronal firing (i.e., after setting the noise parameter *k*_2_ in Eq. (1) to 0): in this case, no episodes of spontaneous CA ignition were observed during the recorded time. These results demonstrate that noise (understood as spontaneous firing) and connection structure critically determine the locus where activation signs emerge first.

## 4 Discussion

We used a neural-network model of primary sensorimotor, secondary, and “higher” association areas in the frontal and temporal lobes to simulate the cortical processes underlying the emergence of action intentions and decisions in the human brain. The model’s behaviour replicates and explains the patterns of activation observed in the brain shortly before initiation of voluntary movement. The ignition of sensorimotor circuits (cell assemblies, CAs) preferentially begins within the model’s higher- association (prefrontal) areas, by way of the spontaneous reverberation of baseline neuronal activity (noise) within the positive-feedback cortical loops that form the CA. Through the CA’s potentiated synapses (which emerged spontaneously, as a result of sensorimotor learning) reverberant activity quickly spreads from prefrontal to premotor areas, reaching the primary motor cortices last, just as observed in *Bereitschaftspotential* experiments investigating the cortical sources underlying voluntary initiation of manual action (Haggard, 2008). In parallel to this sequence of rostro-caudal activations in the frontal lobe, the model exhibits a similar pattern in the temporal lobe, proceeding from polymodal/higher-association areas (posterior–superior or anterior temporal areas, labelled PA in Fig. 1) to secondary (HP), and then to primary (visual or auditory) cortices (P1). Thus, the simulated cortical sources underlying voluntary speech originate simultaneously from inferior prefrontal (Broca’s) and superior temporal (Wernicke’s) areas, subsequently spreading to the adjacent premotor and motor articulatory as well as auditory areas. This reproduces the pattern of neurophysiological activity observed experimentally in tasks where subjects have to generate words, including classic verb generation, sentence completion and auditory verbal imagery tasks (Raichle et al., 1994, Thompson-Schill et al., 1997, Wise et al., 1991) (e.g., see Fig. 1).

These results mechanistically explain the origins and cortical topography of the neural processes underlying endogenously generated intentions and decisions to act in terms of spontaneous, noise-driven ignition of distributed long-term perception–action memory circuits. These ignitions started gradually, with very low – although significantly above baseline – activation values, a stage that we interpret as the model correlate of the (pre-conscious) formation of an initial action *intention*, or preparation (time-steps 0–2). As soon as activation in a perception-action circuit overcomes the “ignition threshold”, it then grows very rapidly, almost “explosively” (time-steps 3–5), outperforming (and suppressing) all its competitor circuits; this can be seen as reflecting a choice, or *decision*, being made between potential alternatives. Note that this account fits very well with recent experimental evidence indicating that decision outcomes of self-initiated actions are already encoded in patterns of brain activity and neuronal firing *several seconds* (up to 10) before they enter awareness (Fried et al., 2011, Soon et al., 2008).

The earliest precursors of, and steepest, activation increases during spontaneous ignitions of perception–action circuits were present within what we refer to as “central”, or higher-association, areas. This local specificity is a direct consequence of the (i) intrinsic network connectivity, and the (ii) neurobiological mechanisms (long-term synaptic plasticity, spreading and reverberation of activation) that constrain the formation of such circuits and determine the way in which activation propagates within them. To see the relevance of the network’s structure, first note that the number of areas projecting to (or receiving projections from) central regions of the model (areas PA, PF) is larger than that for secondary – and primary – ones (see Fig. 1C: each of the central areas is reciprocally linked to *four* other areas, whereas secondary ones to *three*, and primary areas to only *two*). Because at the start of the simulations between-area synaptic links are instantiated at random using the same probability distribution for each pair of areas, neurons in central areas will end up with higher numbers of incoming/outgoing between-area links than cells in secondary (or primary) areas (on average, 4:3:2 ratios for central:secondary:primary, respectively). During training, Hebbian learning acts uniformly across the network, potentiating synapses between any two simultaneously active connected cells. Other things being equal, cells with larger numbers of between-area synaptic links are more likely than others to have such links potentiated. Thus, although learning is driven by concomitant activation of *peripheral* (primary sensory and motor) areas, it is the cells in *central* ones that are more likely to become the constituents of the strongly linked, distributed sensorimotor circuits. This is confirmed by the results, which indicate that more CA cells spontaneously emerged in central than in secondary – and more in secondary than in primary – areas, reflecting the corresponding distribution of the per-area number of “cortico-cortical” (between-area) links. As spontaneous CA ignition is caused by accumulation and reverberation of noise (spontaneous neuronal firing) within the positive-feedback loops that form the CA circuits, and because more between-area potentiated links exist in central than in other areas, under uniform noise, within-CA reverberatory activity is more likely to occur in central than in other areas. (Note that the lead role of higher areas was confirmed even after normalising data for the number of CA cells per area, removing the possibility that the observed phenomena be simply a result of scaling effects.)

The validity of the above explanatory account is backed by two results obtained from additional simulations: first, the observation that, in absence of spontaneous neuronal firing (noise), no spontaneous ignitions occurred during the recorded simulation time (hence, noise is *necessary* to induce reverberation in the perception-action circuits). Second, and most important, the results obtained with a model in which all the “jumping” links (that is, between second-next neighbour areas – purple arrows in Fig. 1C) had been removed. In such a “serially” connected network (containing only the green links Fig. 1C), central and secondary areas have the same average number of incoming/outgoing between-area links (each of them is linked to *two* other areas), a number still larger than that of links to/from primary ones (linked to only *one* other area). We found that, during the early ignition steps, central and secondary areas still became active earlier than primary ones, but, unlike in the “fully connected” model, their activations no longer differed (see Fig. 5, inset). In other words, spontaneous CA ignitions first began in central *and* secondary areas (in equal proportions), while primary areas exhibited significant activity only later, reflecting the new distribution of the per-area number of “cortico-cortical” links (2:2:1). This confirms that the underlying connectivity structure of the model is indeed the factor determining the locus of the origins of spontaneous action thoughts ignitions. In a wider context, these results suggest that the connection structure of prefrontal and temporal higher association cortices may be the explanatory variable of the most intriguing cognitive functions of these regions.

We do not wish to claim here that human volition is *just* the result of the accumulation of neuronal noise within strongly linked sensorimotor circuits. Other mechanisms are certainly necessary to identify which sets of perception–action associations are relevant to the current stimulus, goal or behaviour, and hence are allowed to become active. However, if the decision of *when* to act, or of *which* action to execute out of a set of suitable candidates, is not stimulus driven but “free” and unconstrained, we suggest that the gradual, spontaneous build up of reverberant activity within *distributed* sensorimotor circuits that *compete* with each other to reach full activation may represent an effective way for the cortex to make *both* decisions in a seemingly random (or at least multiply determined) manner. The model explains and demonstrates *how* such decisions can be made by means of processes which (unlike “homunculus”-based ones) are distributed, require only *sub-threshold* (or unconscious) activity, and fall out of well-known neurophysiological principles (spontaneous firing and activation spreading within circuits of reinforced synapses).

Although here we focused on modelling cortical structures and the spontaneous emergence of action intentions within them, we should note that the basal ganglia and cerebellum also play important roles in the preparation and control of action – e.g., (Jueptner & Weiller, 1998). In fact, the presence in the network of a self-regulatory system – realised by area-specific global inhibitory loops (see Section 2.2) – results in a process of mutual competition between the different CA circuits (Garagnani et al., 2008), effectively implementing the action selection functions believed to be mediated by the basal ganglia (Wickens, 1997).

In addition to providing a cortical-level explanation of existing neurophysiological and neuroimaging data on the emergence of voluntary action, the model makes a number of testable predictions. First, the model predicts and explains the spontaneous emergence of perception–action circuits distributed across superior-temporal and inferior-frontal perisylvian areas and linking speech sounds with corresponding articulations (see Fig. 1A); the existence of such action perception circuits in language processing has been confirmed by a number of experimental studies (see (Pulvermüller & Fadiga, 2010) for a recent review). Second, the predicted emergence of sensorimotor circuits distributed across prefrontal–precentral and temporal areas (see Fig. 1B) and mapping specific visual features of objects to specific manual actions has (at least) two further implications: (i) the visual presentation of objects whose features afford (or have been associated to) specific hand/arm movements should elicit activation in prefrontal and premotor areas; and, conversely, (ii) the execution (or mental simulation) of a manual action that has been associated to a particular conjuction of visual features or object should produce, even in *absence* of the associated visual stimulus, activation in *temporal* areas. The former prediction has been confirmed by behavioural (Craighero et al., 1996, Tucker and Ellis, 1998), neuroimaging (Grafton et al., 1997, Grezes and Decety, 2002, Perani et al., 1995) and single cell (Murata et al., 1997, Rizzolatti and Gentilucci, 1988) studies; the latter, novel one, still awaits direct experimental validation, although fMRI and TMS evidence (Cohen et al., 2009, Kroliczak et al., 2007, Singhal et al., 2006) lends some preliminary support.

A growing body of experimental evidence indicates that the observation of goal-directed actions involving hand or mouth movements activates mirror neurons in premotor and parietal regions which are also activated by the execution of the same actions (Fadiga et al., 2005, Fadiga et al., 1995, Gallese et al., 1996, Grafton et al., 1996, Grafton et al., 1997, Rizzolatti et al., 1996a, Rizzolatti et al., 1996b). Note that mirror neuron activation during observation of grasping actions is present only if *both* acting agent (the hand) and target object are visible, which implies the active representation of these visual stimuli and, importantly, of their spatial relationship. This suggests that, in addition to the temporo-frontal perception–action system modelled here and linking information carried by the *ventral* visual stream – specific to object identity – to concordant actions, a second, *parallel* system of sensorimotor brain regions including the parietal areas involved in the processing of the *dorsal* visual stream (Ungerleider & Mishkin, 1982) must be acting in concert with the former to convey visuo-spatial information to hand/arm motor areas (Arbib, 1997, Sakata et al., 1997). Although modelling this second perception–action system was outside the remits of this work, all neurobiological mechanisms simulated here are found pervasively in the cortex; thus, we conjecture that processes of spontaneous sensorimotor circuit emergence and ignition analogous to those simulated in the present model of fronto-temporal areas might occur also in a fronto-parietal (or in any other sensorimotor) system.

Finally, in line with accounts positing the temporary reactivation of long-term cortical representations as a possible neural basis for working memory (Baddeley, 2003, D’Esposito, 2007, Zipser et al., 1993), activation within cells of a CA circuit persists even after withdrawal of the sensory stimulus that elicits it. The discovery of memory cells, neurons exhibiting sustained activity during delayed response tasks (Fuster & Jervey, 1982), is consistent with this behaviour, so the model can, in part, explain the corresponding neurophysiological data. Most importantly, however, this model is the first one to predict that, and explain why, *visuospatial* memory cells are found in *prefrontal* areas (as well as in inferior temporal cortex), as a range of fine experiments unambiguously demonstrated (Funahashi et al., 1989, Fuster, 1990, Kojima and Goldman-Rakic, 1982, Miyashita and Chang, 1988). Note that memory cells have been reported also in primary visual (V1) (Fuster, 1990, Super et al., 2001) and auditory (A1) cortex (Durif et al., 2003, Sakurai, 1994), albeit less frequently there, just as our model predicts (see also (D’Esposito, 2007)).

## 5 Conclusions

We find that the spontaneous emergence of intentions to speak and perform hand-related actions begins in the most “central” parts of the network (PF and PA in Fig. 1, the model correlates of prefrontal and temporal association cortices) due to the underlying between-area connectivity. In fact, in virtue of their rich synaptic links to other areas, these multimodal, higher-association zones (Damasio, 1989) act as “cortical hubs”, linking primary sensory areas to motor ones and thus supporting the formation of distributed perception–action circuits through learning. The hub status of these areas means that their neurons exhibit, on average, a higher number of between-area (cortico-cortical) synaptic links than cells in primary (or secondary) areas, and, hence, are more likely to take part in the formation of perception-action circuits. Subsequently to such circuit formation, in absence of any external input, spontaneous build up and reverberation of noise within their potentiated synapses occurs. Because neuronal noise is uniformly distributed in the network, this is more likely to happen in central areas (where larger numbers of reinforced links have emerged) than in primary or secondary ones.

Solely on the basis of Hebbian learning, cortical connectivity, activation spreading and neuronal noise, our model reproduces and explains the dynamics and cortical topography of the neural processes underlying the spontaneous emergence of decisions to speak and perform manual action. Importantly, the fact that spontaneous activity originates within the most “central” parts of the perception-action circuits mechanistically explains why it is these more richly connected higher-association areas (e.g., prefrontal cortex) that spontaneously take the “lead” over other (primary and secondary) ones, hence naturally becoming the site of processes underlying higher cognitive functions, such as planning and decision making. To the best of our knowledge, this constitutes the first mechanistic model of the spontaneous emergence of *functional specialisation* of brain areas for intentions and decisions, based exclusively on neurobiological principles, anatomical structure and sensorimotor experience.

## Figures and Tables

**Table:** 

Eq. (1)	Time constant (for excitatory cells)	*τ* = 2.5 (in simulation time-steps)
	Time constant (for inhibitory cells)	*τ* = 5 (in simulation time-steps)
	Scaling factor	*k*_1_ = 0.01
	Baseline potential	*V_b_* = 10
	(during training):	*V_b_* = 0
	Noise scaling factor	*k*_2_ = 10√48
	(during training):	*k*_2_ = 25√48
	Global inhibition strength	*k*_S_ = 50
	(during training):	*k*_S_ = 95)
Eq. (3)	Adaptation	*α* = 0.026
Eq. (4.1), (4.2)	Average output time constant (for adaptation mechanism):	*τ*_A_ = 15 (in simulation time-steps)
	Global inhibition time constant	*τ*_S_ = 8 (in simulation time-steps)
Eq. (5)	Postsynaptic potential thresholds for LTP/LTD:	*θ*_−_ = 0.15
		*θ*_+_ = 0.25
	Presynaptic output activity required for any synaptic change:	*θ_pre_* = 0.05
	Learning rate:	Δ*w* = 0.0005

## Footnotes

1 Note that some of the *non*-jumping links in the model (green arrows in Fig. 1C) represent long-distance cortico-cortical connections: namely, those between areas PA and PF, which are adjacent in the model but always represent areas that are not so in neuroanatomical terms.

2 The choice of the number of cells per area is arbitrary and based on empirical evidence: 25-by-25 turns out to be a good compromise between complexity of the system (needed to simulate a sufficiently rich variety of the phenomenon of interest) and total number of cells in the network.

3 Inhibitory cells have *k*_2_ *= *0 (i.e., the neuronal noise is generated just by the excitatory cells).

4 A cell *x* is “activated” by adding the value of 1.0 to the sum of all I/EPSPs that its membrane is receiving in input at that time (i.e., to the term *V_In_*(*x*,*t*) in Eq. (1)). This quickly drives the cell’s membrane potential *V*(*x*) – and, hence, its output *O*(*x*); see Eq. (2) – towards saturation (i.e., 1.0).

5 On the basis of previous data (Garagnani et al., 2008), 1 simulation time-step can be considered equivalent to ∼20 ms.

6 As a cell activated by an external input is given an additional EPSP signal of amplitude 1.0, it is possible to compare directly the amplitude of the noise to that of driven activity. In fact, we know that mean and variance of the noise process *η* are *μ_η_* = 0 and $\sigma_{\eta }^{2}={\left(0.5/\surd 3\right)}^{2}$ , respectively. If we take the signal-to-noise ratio (SNR) to be the ratio of the signal’s mean to the standard deviation of the noise, the SNR of the input signal to a cell which is being excited by an external stimulus is: SNR = (*μ*_signal_/*σ*_noise_) = 1/*σ_η_* = 2·√3 ≈ 3.5.

7 By “CA cells” we mean cells that belong to a cell assembly (CA) circuit (i.e., cells that, by means of Hebbian learning, have become strongly and reciprocally linked to other cells, forming such a circuit).
